# Establishment of Adequate Nutrient Intake Criteria to Achieve Target Weight Loss in Patients Undergoing Bariatric Surgery

**DOI:** 10.3390/nu12061774

**Published:** 2020-06-14

**Authors:** Hee-Sook Lim, Yong Jin Kim, Jihyun Lee, Su-Jin Yoon, Bora Lee

**Affiliations:** 1 Department of Food and Nutrition, Yeonsung University, Anyang 14011, Korea; limhs@yeonsung.ac.kr; 2 Department of General Surgery, H Plus Yang Ji Hospital, Seoul 08779, Korea; 3 Department of Nutrition, H Plus Yang Ji Hospital, Seoul 08779, Korea; dlikej@naver.com; 4 Department of Nutrition, Soonchunhyang University Seoul Hospital, Seoul 04401, Korea; exski@schmc.ac.kr; 5 Department of Statistics, Chung-Ang University, Seoul 06911, Korea; mintbora0125@gmail.com

**Keywords:** bariatric surgery, nutrition, weight loss, caloric restriction, diet

## Abstract

Although bariatric surgery is the best treatment modality for morbidly obese patients, a 10–30% rate of weight recidivism has been reported in various specialized centers. We examined changes in energy and macronutrients after bariatric surgery and performed analysis to establish appropriate nutritional guidelines for reaching the target percentage of weight loss after surgery. A total of 189 subjects who underwent bariatric surgery were classified into success and failure groups depending on whether or not they reached 50% loss of excess weight at 12 months after bariatric surgery. Physical examinations and dietary surveys were completed before and 1, 6, and 12 months after surgery. Using receiver operating characteristic (ROC) analysis, the optimal cutoff points for nutrient intakes for determining success after bariatric surgery were computed based on maximal Youden’s index. At 6 and 12 months after surgery, the success group had significantly lower carbohydrate and fat intakes than the failure group. The cutoff calorie intake for success in weight loss was <835.0, <1132.5, and <1523.0 kcal/day at 1, 6, and 12 months post operation, respectively. With regard to protein, the cutoff intakes were >44.5, >41.5, and >86.5 g/day at 1, 6, and 12 months post operation, respectively. At 12 months, the cutoff ratio for energy obtained from carbohydrates, protein, and fat was <49.0, >24.5, and <28.0%, respectively. Our findings confirm that the level of diet control and nutrition restriction affect the achievement of target weight loss, emphasizing that long-term weight loss is related to compliance with nutrient recommendations.

## 1. Introduction

Bariatric surgery is currently the effective treatment for long-term weight loss and the improvement of obesity-related comorbidities in morbidly obese patients [1]. The most significant advantage of the surgical procedure is a large weight loss effect achieved by reduced food intake and malabsorption, and this weight loss can be maintained in the long term [2]. However, side effects can cause various changes in the intestine after surgery and malnutrition due to the reduced dietary intake [3].

Patients are encouraged to receive nutritional management before and after bariatric surgery as well as nutritional education from experts, including blood tests and nutritional assessments [4]. In general, bariatric surgery is considered successful if more than 50% of the excess weight is lost over 2 years [5]. A poor-quality diet, lack of physical activity, and lack of continuous nutritional management are the main reasons for failure, along with excessive energy consumption and overeating after surgery [6,7]. Therefore, assessment of patients’ eating patterns and individualized nutritional management are critical for successful long-term weight loss.

Surgical procedures are effective for successful weight loss, but nutrient intake after surgery also plays a key role in achieving the long-term goals [2,4]. The recommended percentage of energy intake from carbohydrates after bariatric surgery is 35–48%, with an intake of at least 60–80 g protein/day [8,9,10]. There have been a number of studies on calorie intake and nutrient deficiency, but the results in relation to weight loss vary [3,8]. In addition, the specific criteria guidelines for proper nutrient intake according to the surgical overtime point are insufficient. In Korea, proper standards for carbohydrate-based meals need to be set, as there is only a broad guideline of 1000–1400 kcal/day after surgery [11].

This study was performed to explore the relationship between energy intake after bariatric surgery and weight loss, and to establish appropriate nutritional guidelines to reach the target percentage of weight loss after surgery.

## 2. Materials and Methods

### 2.1. Subjects

This retrospective observational study used data from a single center. The eligibility criteria for bariatric surgery were an age of 20–65 years with a BMI ≥ 35 kg/m^2^ or BMI ≥ 30 kg/m^2^ and a comorbidity related to obesity [12]. All eligible subjects underwent bariatric surgery between April 2013 and January 2018, and all the surveys and management were conducted at each visit. Subjects with absence of data on diet or weight loss after surgery and those who underwent hospitalization or reoperation due to complications after surgery, were excluded. A total of 189 patients were included in the analysis. The study protocol was approved by the Institutional Review Board of Soon Chun Hyang University Seoul Hospital (IRB number: SCH IRB 2019-02-006; approval date: 7 March 2019). Written informed consent was obtained from all patients. All subjects voluntarily agreed to participate in the study after receiving a detailed description of the procedures and goals. The study protocol conformed to the ethical guidelines of the World Medical Association Declaration of Helsinki.

### 2.2. General Characteristics and Anthropometric Measurements

The baseline demographics of the enrolled subjects—including age, sex, body mass index (BMI), and lifestyle habits (alcohol drinking, smoking, exercise, and experience with diet control)—at the time of the operation and anthropometric data at all follow-up time points were collected from the metabolic and obesity surgery database and hospital medical records. Measurements of body composition were performed at each follow-up time with a bioelectrical impedance analyzer (Biospace In-body 720, Seoul, Korea). Using the checked height and weight, we calculated the body mass index (BMI, kg/m^2^). Fat free mass (FFM) was calculated by subtracting fat mass from body weight (FFM = Body weight − (Body weight × Fat%)). %Excess weight loss (%EWL) was calculated using the formula (weight loss/baseline excess weight) × 100, where weight loss = preoperative weight−initial weight; baseline excess weight = initial weight−ideal weight (X), and X = 23 × height (m)^2^. X was calculated using an ideal BMI, as the ideal BMI cutoff point has been demonstrated to be 23 kg/m^2^ [13]. All subjects were classified into success and failure groups based on %EWL 50% at 12 months after bariatric surgery.

### 2.3. Dietary Intake Analysis

Our institute has a protocol for nutritional management procedures about for nutritional assessments, nutritional education, and counseling—preoperatively and at 1, 3, 6, and 12 months post operation via interviews between the individual patient and a clinical dietitian. The clinical dietitian calculated the average daily nutrient intake based on 3-day food records for all foods and drinks. The consumed food was analyzed using Computer Aided Nutritional Analysis Program for Professionals 4.0 (CAN-Pro 4.0, The Korean Nutrition Society, Korea) that analyzes the intake of individual nutrients.

### 2.4. Follow-Up

Subject’s data were obtained preoperatively and at 1, 6, and 12 months post operation. Changes in anthropometric status and nutrient intake were analyzed at each visit.

### 2.5. Statistical Analyses

Power calculation was conducted to determine the sample size required to detect the clinical effect via PASS (ver 12. NCSS, LLC. Kaysville, Utah, USA; www.ncss.com). It was figured out that 127 subjects from the success group and 62 from the failure group provided 93 to 100% power to detect a difference in the range of 0.0310 to 0.1620 between the areas under the ROC curve (AUCs) of the calorie, carbohydrate, and fat intakes at 12 months post operation and the null hypothesis; and an AUC of 0.75, which has clinical meaning as a diagnostic tool; while the two-sided z-test was set at a significance level of 0.05. The demographic characteristics and clinical factors of the subjects are presented as means ± standard deviation for continuous variables and as frequencies with percentages for categorical variables. Success and failure groups were compared for the change in amount of body weight, the proportion of the categorized %EWL, and the nutrition intakes at each follow-up time. Wilcoxon’s rank sum test was used for comparing the continuous variables and the χ^2^ test or Fisher’s exact test were used for comparing the categorical variables as appropriate. The associations between %EWL and macronutrient intake levels at 12 months post operation were evaluated by Pearson’s method. Logistic regression analysis was performed for factors affecting %EWL success. Using receiver operating characteristic (ROC) analysis, the optimal cutoff points of nutrient intakes for determining success after bariatric surgery were computed based on maximal Youden’s index. The sensitivity, specificity, accuracy, positive likelihood ratio (LR+), and negative likelihood ratio (LR−) of the successful weight loss are presented with 95% confidence intervals (CIs), which were calculated by exact binomial distribution for sensitivity, specificity, and accuracy and a formula provided by Simel et al. [14] for positive and negative likelihood ratios. All analyses were carried out using R (version 3.6.3; The R Foundation for Statistical Computing, Vienna, Austria), and a two-sided *p*-value of less than 0.05 was considered to indicate statistical significance.

## 3. Results

### 3.1. Clinical Characteristics and Weight Loss

The general characteristics and weight loss results of the 189 subjects are shown in Table 1 and Table 2, respectively. The average age was 34.6 years, and 71.4% of them were female. The surgical method was laparoscopic Roux-en-Y gastric bypass (LRYGB) in 77.2% and sleeve gastrectomy (SG) in 22.8% of subjects. The main comorbidities included diabetes, dyslipidemia, and hypertension. The rates of lifestyle habits before surgery were as follows: alcohol drinking, 34.9%; smoking, 46%; exercise, 14.8%; and experience with diet control, 46.0%. The average BMI was 38.9 ± 5.9 kg/m^2^, and the amount of excess weight was 44.2 ± 16.6 kg. The average %EWL at 1, 3, 6, and 12 months post operation were 24.69%, 41.66%, 46.42%, and 53.05%, respectively. Whereas the success group had an average %EWL of 52.76% at 6 months, the %EWL was delayed by 6 months in the failure group, and the %EWL was 37.46% at 12 months. The average %EWL according to the operative method and comorbidity showed no significant differences.

### 3.2. Changes in Nutrient Intake over Time

The changes in nutritional intake before and after surgery are shown in Table 3. There were no significant differences in the intake of macronutrients or calories before surgery between the two groups. At 1 month post operation, the calorie intake was about 760 kcal in two groups, and the protein intake was 58.02 g in the success group and 50.55 g in the failure group. At 6 months, the calorie intake was 999.82 kcal in the success group (*p* = 0.004), and the carbohydrate (*p* < 0.001) and fat intakes (*p* = 0.022) were significantly lower in the success group. At 12 months, the calorie intake was 1336.75 kcal in the success group and 1646.21 kcal in the failure group (*p* < 0.001). Comparing the energy ratios of macronutrients, the carbohydrate ratio was significantly lower at 1 month (*p* = 0.031) and 12 months (*p* = 0.004) in the success group, the protein ratio was significantly higher at 6 months (*p* < 0.021) and 12 months (*p* = 0.010) in the success group, and the fat ratio was significantly lower at 12 months (*p* = 0.040) in the success group.

### 3.3. Correlation between %EWL and Nutrient Intake

**Figure 1:**
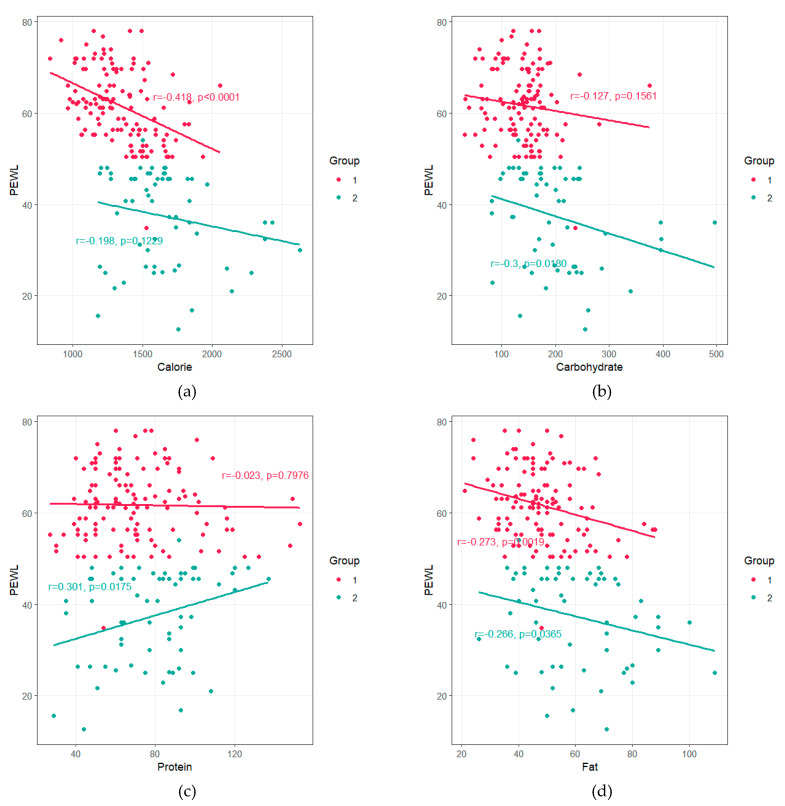
Scatterplot representing the association between %EWL and macronutrient composition at postop 12 months. (**a**) correlation between %EWL and calorie, (**b**) correlation between %EWL and carbohydrate, (**c**) correlation between %EWL and fat, (**d**) correlation between %EWL and fat. Group 1; Success, Group 2; Failure. *P*-value was computed by pearson correlation test.

The correlations between %EWL and nutrient intake at 12 months post operation are shown in Figure 1. The %EWL in the success group was inversely related to calorie (*r* = −0.418, *p* < 0.0001) and fat intakes (*r* = −0.273, *p* = 0.0019). In the failure group, the %EWL showed inverse relationships with carbohydrate (*r* = −0.3, *p* = 0.018) and fat intakes (*r* = −0.266, *p* = 0.0365), while it showed a positive correlation with protein intake (*r* = 0.301, *p* = 0.0175).

### 3.4. Factors Affecting the %EWL in Subjects

The logistic regression analysis results for factors affecting %EWL at 12 months post operation are shown in Table 4. In the univariable logistic regression, significant effects were confirmed for age, calories at 6 and 12 months, carbohydrates at 6 and 12 months, protein at 1 and 12 months, fat at 6 and 12 months, and all the proportions of calories at 12 months versus the three components. To avoid the multicollinearity in the regression analysis, the final model was created including age and the calorie proportion versus the three components. It shows an insignificant effect for the proportion of calories at 12 months versus protein (OR, 0.99; 95% CI, 0.97% to 1.00%). Excluding this factor, the final model has the factors affecting %EWL at 12 months as follows: lower age (OR, 0.96; 95% CI, 0.93% to 0.99%), lower proportion of calories as carbohydrates (OR, 0.99; 95% CI, 0.98% to 0.99%), and proportion of calories as fat (OR 0.96; 95% CI, 0.93% to 0.98%).

### 3.5. Optimal Nutrient Intakes for Determining Success after Bariatric Surgery

The optimal cutoff point for nutrient intakes for determining success after bariatric surgery at each postoperative time point are shown in Table 5. The cutoff calorie intakes were <835.0, <1132.5, and <1523.0 kcal/day at 1, 6, and 12 months post operation, respectively. With regard to protein, the cutoff intakes were >44.5, >41.5, and >86.5 g/day at 1, 6, and 12 months post operation, respectively. At 12 months, the cutoff percentages for calories obtained from carbohydrates, protein, and fat were <49.0%, >24.5%, and <28.0%, respectively.

## 4. Discussion

Bariatric surgery is considered the best treatment for clinically severe obesity, which can result in long-term body weight loss and the control or remission of comorbidities [15]. Roux-en-Y gastric bypass (RYGB) and sleeve gastrectomy (SG) are the most common procedures used in bariatric surgery [16]. Despite its advantages, bariatric surgery may compromise nutritional status due to food intake restriction [17]. The greatest decrease in food intake occurs within the first 3 months post operation, so regular follow-ups with a clinical dietitian are important for facilitating adequate weight loss and preventing malnutrition [18,19].

Nutrition prescription is an important component of nutritional interventions for weight management. In particular, calorie prescription is the key for guiding intake amounts [20]. Over time, energy intake shows an increasing trend, with values of 1500, 1700, 1800, 1900, and 2000 kcal/day at 6 months and 1, 2, 3, and 4–10 years post operation [9]. Several studies have reported weight loss effects according to calorie restriction [18,21]. A very-low-calorie diet was shown to induce greater changes compared with a low-calorie diet [22]. Other studies that evaluated body nutrient composition changes following a short-term low-calorie diet of 1500 kcal/day indicated that lean body mass accounted for 31.0–38.6% of total weight loss during the study period [23,24]. It can be quite challenging to determine the energy requirements for patients after bariatric surgery. Predictive equations are commonly used for requirement calculations, but their accuracy can vary, and they do not take into consideration body composition or usual dietary intakes, which can affect energy requirements. In this study, we found a significant difference in energy intake at 6 months after surgery, and weight loss success showed a significant correlation with energy intake. The energy intake was 835 kcal at 1 month after surgery, and maintaining a very-low-calorie or low-calorie diet until 1 year can help with reaching the weight loss goal. However, in order to maintain a long-term weight loss, a balanced intake of nutrients should be demonstrated and there should be no nutritional deficiency, rather than simply depending on total calorie intake.

There are no precise recommendations for carbohydrate intake after surgery, but 130 g of carbohydrates/day provides sufficient glucose for the central nervous system [25]. Das et al. [26] suggested that a meal pattern with a high glycemic load represents an increase of 29% in energy intake compared with a meal pattern with a low glycemic load. Another study suggested that the intake of a high glycemic load is associated with a higher calorie intake and less weight loss [27]. Most studies recommended avoiding simple sugars and choosing foods rich in complex carbohydrates and dietary fiber with a low glycemic index [28]. In the present study, we could not analyze the types of starch in the diet, but carbohydrate intake showed a significant difference at 6 months postoperatively between two groups, and we instructed patients to maintain carbohydrate consumption within 173 g/day until 1 year post operation. This cutoff value was slightly higher than the values reported in other countries. According to the 2018 Korea National Health and Nutrition Survey, the recommended carbohydrate intake for adults is 294 g/day, contributing 66% of total energy [29]. Rice is the staple food in Korea, and the Korean diet is based mainly on carbohydrates. Although the amount of meals increased over time during the postoperative period, carbohydrate intake was strictly restricted until 6 months in our results.

Protein intake promotes rapid postoperative recovery and helps maintain muscle mass [19]. In the American Society for Metabolic and Bariatric Surgery (ASMBS), the recommended protein requirements are 60–80 g/day or 1.5 g per ideal body weight to preserve body protein levels [10]. However, it is practically not easy to meet the recommended amount. Foods that are sources of protein contain fat, and so it is necessary to eat low-fat protein sources or use an oral protein supplement. The protein intake at 1 month in the success group was significantly higher compared with that in the failure group, with a protein intake exceeding 86.5 g/day at 12 months. However, compared with other nutrients, the cutoff value for protein intake had low accuracy in terms of predicting success after bariatric surgery.

Finally, the results regarding the percentage of energy obtained from macronutrients were notable. Many studies have reported the following postoperative macronutrient distributions: 35–50% from carbohydrates, 15–23% from protein, and 35–42% from fat [4,9,10]. Swenson et al. [30] reported that subjects to either a low-fat diet or a low-carbohydrate, high-protein diet for 1 year showed significant weight loss both by a reduction in BMI and excess body weight loss. At 12 months, the recommended proportions of energy obtained from carbohydrates, protein, and fat are <49%, >24.5%, and <28%, respectively; the proportion of carbohydrate is especially meaningful.

This study had some limitations. Obese patients tend to underreport their dietary intakes, and therefore, the estimated energy intake may have been lower than the actual intake. Nutritional deficiencies in the subjects were not analyzed, the observation period was short, and sample size was small. It did not reflect, as a variable, whether the time and intensity of the exercise affected success. The percentage of failure group in all subjects was reported to be high at 32.8%. The percentages of subjects in the total study population with %EWL < 25% were 4.2% at 1 month, 4.2% at 6 months, and 3.2% at 12 months, although these data are not presented.

A major strength of this study was that the data were detail-managed by professional dietitians. In addition, criteria for proper macronutrient intake were guided at each follow-up point after surgery, providing basic data for future nutritional guidelines. Even though the area under the curve (AUC) values did not seem to be large enough to find a good optimal cutoff point, the attempt was meaningful in establishing nutrient intake standards based on the data.

## 5. Conclusions

In conclusion, based on the data obtained at 12 months after bariatric surgery, carbohydrate and fat intake are significantly related to weight loss. Adhering to adequate nutrient intake during the postoperative period appears to be an effective treatment for morbid obesity. Further investigations are required to determine the long-term effects of bariatric surgery on weight loss, nutrient intake, and nutritional status.

## Figures and Tables

**Table 1:** Baseline characteristics in all patients.

Variable	Total (*n* = 189)
Age (years)	34.6 ± 10.7
Sex	
Male	54 (28.6%)
Female	135 (71.4%)
Operative method	
LRYGB	146 (77.2%)
SG	43 (22.8%)
Comorbidity	
Yes	43 (22.8%)
Diabetes	18 (9.5%)
Hypertension	10 (5.3%)
Hyperlipidemia	11 (5.8%)
Joint problem	6 (3.2%)
Depression	4 (2.1%)
Apnea	7 (3.7%)
Number of comorbidities	
One	26 (60.4%)
Two	11 (25.6%)
Three or more	6 (14.0%)
Lifestyle habit	
Alcohol drinking	66 (34.9%)
Smoking	36 (19.0%)
Exercise	28 (14.8%)
Experience of diet control	86 (46.0%)
Weight (kg)	108.0 ± 19.8
BMI (kg/m^2^)	38.9 ± 5.9
Excess weight (kg)	44.2 ± 16.6
Fat Mass (kg)	55.7 ± 11.3
Fat Free Mass (kg)	51.6 ± 13.9

LRYGB, Roux-en-Y gastric bypass; SG, sleeve gastrectomy. Data are reported as mean ± SD for continuous variables and *n* (%) for categorical variables.

**Table 2:** Changes in percentage of excess weight loss (%EWL) between two groups.

Variable	Total (*n* = 189)	Achievement of Weight Loss			Operative Method			Comorbidity		
		Success (*n* = 127)	Failure (*n* = 62)	*p*-Value	LRYGB (*n* = 146)	SG (*n* = 43)	*p*-Value	Yes (*n* = 43)	No (*n* = 146)	*p*-Value
Postop 1 month	24.69 ± 9.23	25.85 ± 10.00	22.50 ± 6.52	0.005	24.52 ± 9.66	25.24 ± 7.64	0.615	24.59 ± 7.82	25.25 ± 13.39	0.695
Postop 3 months	41.66 ± 11.57	45.38 ± 10.87	34.03 ± 8.96	<0.001	42.18 ± 12.91	41.50 ± 11.19	0.736	45.35 ± 16.06	48.71 ± 17.57	0.258
Postop 6 months	46.42 ± 17.27	52.76 ± 17.12	33.48 ± 7.78	<0.001	50.05 ± 18.66	46.67 ± 16.75	0.289	41.98 ± 10.87	42.24 ± 13.58	0.900
Postop 12 months	53.05 ± 15.90	61.71 ± 7.90	37.46 ± 10.21	<0.001	54.40 ± 16.38	53.57 ± 13.75	0.762	53.15 ± 14.80	54.81 ± 13.81	0.511

LRYGB, Roux-en-Y gastric bypass; SG, sleeve gastrectomy. Data are reported as mean ± SD and *p*-values were calculated with Wilcoxon’s rank-sum test.

**Table 3:** Comparison of nutrition intakes between two groups.

Variable	Calorie (kcal)			Carbohydrate (g)			Protein (g)			Fat (g)		
	Success (*n* = 127)	Failure (*n* = 62)	*p*-Value	Success (*n* = 127)	Failure (*n* = 62)	*p*-Value	Success (*n* = 127)	Failure (*n* = 62)	*p*-Value	Success (*n* = 127)	Failure (*n* = 62)	*p*-Value
Preop	2282.97 ± 626.93	2234.31 ± 609.10	0.610	293.31 ± 90.00 (53.5%)	311.18 ± 113.16 (56.4%)	0.280	92.06 ± 32.42 (16.8%)	84.02 ± 28.75 (15.1%)	0.086	74.24 ± 32.67 (29.9%)	70.77 ± 31.61 (28.5%)	0.485
Postop 1 month	769.33 ± 217.88	765.73 ± 178.30	0.904	70.48 ± 34.82 (36.7%)	70.56 ± 35.47 (39.6%) ^a^	0.987	58.02 ± 21.95 (30.1%)	50.55 ± 23.74 (28.9%)	0.040	30.04 ± 14.53 (33.2%)	27.59 ± 14.25 (31.5%)	0.272
Postop 6 months	999.82 ± 259.30	1120.81 ± 272.43	0.004	97.58 ± 44.1 (40.6%)	133.98 ± 54.96 (46.4%)	<0.001	57.87 ± 20.91 (25.4%)	53.79 ± 18.06 (20.2%) ^b^	0.169	34.71 ± 11.53 (34.0%)	38.98 ± 12.10 (33.4%)	0.022
Postop 12 months	1336.75 ± 229.03	1646.21 ± 315.55	<0.001	139.13 ± 49.04 (48.4%)	198.60 ± 81.10 (53.1%) ^A^	<0.001	79.19 ± 24.09 (28.0%)	70.43 ± 25.04 (20.4%) ^B^	0.023	47.87 ± 12.32 (23.6%)	59.42 ± 17.53 (26.5%) ^C^	<0.001

Data are reported as mean ± SD. *P*-values were computed by Wilcoxon’s rank-sum test. Numbers of patients were 127 for success and 62 for failure. Data in parentheses report the average energy ratio for each macronutrient intake. (^a^ *p* < 0.05 vs. carbohydrate in postop 1 month, ^A^ *p* < 0.01 vs. carbohydrate in postop 12 months, ^b^ *p* < 0.05 vs. protein in postop 6 months, ^B^ *p* < 0.05 vs. protein in postop 12 months, ^C^ *p* < 0.05 vs. fat in postop 12 months).

**Table 4:** Logistic regression analysis for the success in patients.

Variable	Univariable		Multiple 1		Multiple 2	
	OR (95% CI)	*p*-Value	OR (95% CI)	*p*-Value	OR (95% CI)	*p*-Value
Age (year)	0.96 (0.93–0.99)	0.009	0.97 (0.94–0.99)	0.046	0.96 (0.93–0.99)	0.027
Female	1.83 (0.94–3.52)	0.072				
LRYGB	0.74 (0.34–1.54)	0.437				
No. of comorbidities	0.85 (0.65–1.13)	0.249				
Calorie (100 kcal)						
at 1 month	1.05 (0.91–1.22)	0.515				
at 6 months	0.78 (0.68–0.88)	<0.001				
at 12 months	0.37 (0.27–0.48)	<0.001				
Carbohydrates (10 g)						
at 1 month	0.98 (0.9–1.07)	0.607				
at 6 months	0.85 (0.78–0.91)	<0.001				
at 12 months	0.63 (0.54–0.72)	<0.001				
Protein (10 g)						
at 1 month	1.17 (1.01–1.36)	0.038				
at 6 months	0.99 (0.85–1.15)	0.853				
at 12 months	0.85 (0.74–0.96)	0.008				
Fat (10 g)						
at 1 month	1.11 (0.9–1.39)	0.323				
at 6 months	0.55 (0.41–0.72)	<0.001				
at 12 months	0.46 (0.35–0.6)	<0.001				
Proportion of calories at 12 months						
Carbohydrate (%)	0.98 (0.98–0.99)	<0.001	0.98 (0.98–0.99)	<0.001	0.99 (0.98–0.99)	<0.001
Protein (%)	0.99 (0.97–1.00)	0.026	0.99 (0.97–1.00)	0.171		
Fat (%)	0.95 (0.93–0.97)	<0.001	0.96 (0.93–0.99)	0.002	0.96 (0.93–0.98)	<0.001

OR, odds ratio; CI, confidence interval.

**Table 5:** Prognostic performance for the success in patients.

Variable	AUC (95% CI)	Optimal Cutoff *	Estimates (95% CI)				
			Sensitivity	Specificity	Accuracy	LR (+)	LR (−)
Calories (kcal)							
at 1 month	0.529 (0.442–0.615)	<835.0	0.57 (0.48–0.65)	0.32 (0.21–0.45)	0.49 (0.41–0.56)	0.84 (0.67–1.05)	1.34 (0.89–2.03)
at 6 months	0.673 (0.593–0.754)	<1132.5	0.75 (0.66–0.82)	0.55 (0.42–0.68)	0.68 (0.61–0.75)	1.66 (1.24–2.22)	0.46 (0.32–0.67)
at 12 months	0.912 (0.872–0.953)	<1523.0	0.87 (0.79–0.92)	0.82 (0.7–0.91)	0.85 (0.79–0.90)	4.88 (2.84–8.38)	0.16 (0.10–0.26)
Carbohydrate (g)							
at 1 month	0.513 (0.424–0.602)	<115.5	0.91 (0.85–0.96)	0.15 (0.07–0.26)	0.66 (0.59–0.73)	1.07 (0.95–1.20)	0.60 (0.26–1.36)
at 6 months	0.703 (0.624–0.781)	<103.0	0.58 (0.49–0.67)	0.79 (0.67–0.88)	0.65 (0.58–0.72)	2.78 (1.68–4.61)	0.53 (0.41–0.67)
at 12 months	0.878 (0.819–0.937)	<172.5	0.93 (0.87–0.97)	0.76 (0.63–0.86)	0.87 (0.82–0.92)	3.84 (2.47–5.98)	0.09 (0.05–0.18)
Protein (g)							
at 1 month	0.617 (0.529–0.705)	>44.5	0.76 (0.67–0.83)	0.52 (0.39–0.65)	0.68 (0.61–0.74)	1.56 (1.19–2.06)	0.47 (0.32–0.70)
at 6 months	0.524 (0.440–0.609)	>41.5	0.86 (0.79–0.91)	0.02 (0.00–0.09)	0.58 (0.51–0.65)	0.87 (0.81–0.94)	8.79 (1.02–64.33)
at 12 months	0.618 (0.531–0.705)	>86.5	0.21 (0.15–0.29)	0.55 (0.42–0.68)	0.32 (0.26–0.39)	0.47 (0.31–0.73)	1.44 (1.13–1.83)
Fat (g)							
at 1 month	0.564 (0.475–0.652)	<21.5	0.49 (0.38–0.61)	0.58 (0.48–0.67)	0.54 (0.47–0.62)	0.58 (0.4–0.83)	1.45 (1.09–1.92)
at 6 months	0.682 (0.599–0.766)	<46.5	0.85 (0.78–0.91)	0.48 (0.35–0.61)	0.73 (0.66–0.79)	1.65 (1.28–2.12)	0.31 (0.19–0.50)
at 12 months	0.781 (0.709–0.853)	<52.5	0.78 (0.70–0.85)	0.69 (0.56–0.8)	0.75 (0.68–0.81)	2.54 (1.73–3.74)	0.32 (0.22–0.46)
Proportion of calorie at 12 months							
Carbohydrate (%)	0.714 (0.637–0.792)	<49.0	0.85 (0.78–0.91)	0.60 (0.46–0.72)	0.77 (0.7–0.83)	2.11 (1.54–2.88)	0.25 (0.16–0.40)
Protein (%)	0.609 (0.523–0.695)	>24.5	0.52 (0.40–0.64)	0.74 (0.65–0.81)	0.65 (0.58–0.72)	1.98 (1.36–2.88)	0.65 (0.50–0.84)
Fat (%)	0.855 (0.792–0.917)	<28.0	0.73 (0.65–0.81)	0.60 (0.46–0.72)	0.69 (0.62–0.75)	1.82 (1.32–2.50)	0.45 (0.32–0.64)

CI, confidence interval; AUC, area under the curve; LR, likelihood ratio. * based on Youden’s index.
